# A fine-grained time course investigation of brain dynamics during conflict monitoring

**DOI:** 10.1038/s41598-019-40277-3

**Published:** 2019-03-06

**Authors:** Paolo Ruggeri, Hadj Boumediene Meziane, Thomas Koenig, Catherine Brandner

**Affiliations:** 1 Brain Electrophysiology Attention Movement Laboratory, Institute of Psychology, University of Lausanne, Lausanne, Switzerland; 2 Translational Research Center, University Hospital of Psychiatry, University of Bern, Bern, Switzerland

## Abstract

The conflict monitoring model predicting higher anterior cingulate cortex (ACC) neuronal activity on incongruent trials has been recently challenged by a model predicting longer neuronal activity in incongruent trials characterized by longer RTs. To clarify this issue, brain dynamics were explored through event-related-potential (ERP) recordings during a Stroop task. We assessed differences between experimental conditions by combining complementary methods sensitive to the temporality of events including microstate, TANOVA and source localization analysis. The analysis demonstrated the same electrical dynamics only differed in duration towards the end of information processing in the incongruent condition. Specifically, the activation strength of the ACC region did not differ significantly between congruent and incongruent conditions but lasted longer in the incongruent condition. Taken together, our results support the model predicting longer neuronal activity in incongruent trials characterized by longer RTs. They highlight that brain dynamics can dramatically change through periods of interest and that caution is required when interpreting fMRI results. To conclude, these results indicate how time-sensitive measures can contribute to a better understanding of the mechanisms underlying information processing, and thus offer new venues to explore conflict monitoring

## Introduction

Efficient information processing requires both rapid selection and inhibition of information to behave appropriately to a given situation. Rapid selection requires flexible adaptation, relying on a smooth engagement and disengagement of attentional focus. In this context, attention operates through a set of filters, or weights, that are deployed to bias information processing to extract information relevant to the current task goals^1^. The mechanisms supporting such adaptability, referred to as attentional cognitive control, have been the focus of growing research encompassing different domains, from behavioral to cognitive neuroscience^2^. Different theoretical frameworks focus on parsing subcomponents of attentional control and their underpinning neural substrates^3–6^. Among them, an influential model of attentional control, referred to as the conflict monitoring model^7–15^, proposes that conflicting information (e.g., a mismatch between incompatible responses, or a mismatch between current goals and outcomes) is controlled by specialized regions in the anterior cingulate cortex (ACC), which mediate decision-making processes that subsequently trigger reallocation of attentional resources through activation of dorsolateral prefrontal cortex (DLPFC) regions.

The conflict monitoring model is grounded on behavioral and neuroimaging results from paradigms using conflicting stimulus inputs, such as the Stroop task^16,17^ or the Eriksen flanker task^18^. These tasks display series of visual stimuli with task-relevant and task-irrelevant stimulus features that can either be in conflict to each other (e.g., incongruent) or not (e.g., congruent). In the Stroop task, for example, subjects are typically asked to name the ink color used to print the displayed color word. In the congruent condition, the task-relevant (ink color) and task-irrelevant (written word) features correspond to each other (e.g., “RED” presented in red ink), while they differ in the incongruent condition (e.g., “RED” presented in green ink). The decrease in behavioral performance observed during incongruent stimulus processing is indicated by slower response times (RTs) and increased number of errors compared to congruent stimulus processing^13,17,19,20^. Numerous neuroimaging studies contrasting neural responses to congruent and incongruent visual stimulus inputs found differential activation of the ACC. Using positron emission tomography (PET), Pardo and colleagues^21^ were the first to show increased ACC activation during incongruent stimulus processing as compared to congruent stimulus processing and this finding was supported by following PET^22–24^ and fMRI^7,10,11,13,14,25–32^ studies.

Further research using electroencephalographic (EEG) techniques was intended to provide a detailed description of the temporal evolution of event-related potentials (ERPs) recorded during tasks involving conflicting stimuli. In the Stroop task, for example, an ERP component, peaking between 400 and 600 ms, was consistently observed after the incongruent stimulus onset. This component, referred as N_inc_, is characterized by a more negative electrical potential over frontal-medial scalp locations in the incongruent compared to the congruent condition^33–42^. In line with the neuroimaging results, source imaging modelling identified the ACC regions as the neural generator of this component^20,35,36^. The relatively early onset of ACC activation differences revealed by ERP studies provided further support for the ACC involvement in at least one aspect of conflict monitoring^8^.

Recent fMRI studies using conflict paradigms have provided alternative interpretations to that of the conflict monitoring model. The authors^43–46^ point out that the difference in the duration to process congruent and incongruent stimuli could have a significant impact on the recorded hemodynamic response. This is an important point as it is as yet unclear whether the observed activation of ACC regions reflects the amount of response conflict or the time needed by participants to release the correct motor response. Grinband and colleagues^47^ used event-related fMRI recording during a Stroop paradigm to tackle this key issue. Their findings showed that the ACC BOLD activation was not sensitive to the processing of conflicting stimuli, but rather correlated to RT. Although higher activation of the ACC regions in the incongruent condition than in the congruent one was found, higher activation was also found when slow RT congruent trials were contrasted to fast RT incongruent trials. Also, comparable activation was recorded in both congruent and incongruent trials characterized by comparable RT lengths. Taken as a whole, these findings reinforced doubts about the conflict monitoring role previously attributed to the ACC. They suggested instead a larger involvement of the ACC regions supporting a variety of processes, such as those related to autonomic arousal or cognitive effort supposed to be engaged as long as a motor response is executed. Therefore, the additional theoretical framework predicting longer engagement of neuronal activity on incongruent trials characterized by longer RTs^47^ appears to be a robust alternative to the conflict monitoring model which predicts higher ACC neuronal activity per unit time on incongruent trials^8^. However, given the limited temporal resolution of fMRI, only indirect evidence has been provided in support to this alternative, thus leaving this question open to further debate^44,48^.

Recording EEG activity would help to clarify the role of the ACC by providing a direct, fine time-scale description of the dynamics of the underlying neural population activity. Unfortunately, most ERP studies, including those reviewed above, have not fully exploited the amount of spatiotemporal information contained in EEG recordings. For example, comparisons between congruent and incongruent conditions at predefined time points or temporal windows, and often at selected electrode locations, provided valid but incomplete evidence about why participants took longer to respond in the incongruent condition.

To investigate possible differences between experimental conditions, and provide further evidence to the computational model proposed by Grinband and colleagues^47^, we choose a more suitable alternative solution for characterizing ERPs. We used a temporal sequence of electrical potential field topographic maps for describing ERPs traces, combined with complementary methods sensitive to the temporality of events, to explore brain dynamics recorded during a Stroop task.

In our paradigm, the task-relevant stimulus feature (i.e., the ink color or the written word) was cued to the participants on a trial-by-trial basis before the presentation of a color-word stimulus. This resulted in four different experimental conditions: (i) color-congruent condition (cC), e.g., name the ink color of the word RED printed in red ink; (ii) color-incongruent condition (cI), e.g., name the ink color of the word RED printed in green ink; (iii) word-congruent condition (wC), e.g., read the written word of the word RED printed in red ink; (iv) word-incongruent condition (wI), e.g., read the written word of the word RED printed in green ink. To assess differences between experimental conditions, we used a microstate analysis approach^49,50^ combined with reference independent topographic analysis^51,52^ allowing classifying ERP dynamic changes in a set of stable topographical map configurations. Each of the microstate maps represents the electrical potential activity generated by the activation of different large-scale neuronal networks. This method also provides tools for assessing differences in onset, offset, duration, and strength of activation of each microstate map related to experimental conditions^50,53^. To ensure comparison with fMRI studies, we used source localization^54^ as a mean to estimate neural generators underlying topographic differences between experimental conditions.

Consistent with the results of previous studies^55,56^, microstate analysis should identify the same number of brain processes, occurring in the same temporal sequence, for both congruent and incongruent experimental conditions. However, in accordance with the computational model proposed by Grinband and colleagues^46^, the duration of late processes in the incongruent condition of the Stroop task supposed to reflect conflict resolution should be extended. On this basis, we predict that the global topographic analysis will reveal significant topographic differences between congruent and incongruent conditions due to a time-lag in the succession of stable microstates. In line with what was predicted at the scalp level, we do not expect topographic differences due either to different cortical generators active at the same time, or to similar generators whose activity differs at the source level. We assume instead the involvement of similar cortical generators in both the congruent and incongruent conditions, but with different synchronizations between conditions.

## Materials and Methods

### Participants

Thirty-eight healthy volunteers (19 females; mean age = 23.5 years, SD = 8.1 years, range 18 to 50 years) gave written informed consent to participate to the experiment and were compensated with credits for their participation. All participants reported normal or corrected-to-normal vision acuity and a right-hand preference. Research was approved by the Cantonal Ethics Committee for Human Research (Vaud, Switzerland; protocol N°286/13) and was in accordance with the code of ethics of the World Medical Association (Declaration of Helsinki) for experiments involving human subjects in research.

### Experimental Procedure

During the testing session participants were comfortably seated in a moderately dark room (Faraday cage) facing a monitor placed at 60 cm from their eyes while performing a Stroop task. The Stroop task consists in the presentation of congruent and incongruent color-word stimuli. In the congruent condition, the two features of the color-word stimuli (written word and ink color) coincide, while they differ in the incongruent condition. Each trial of the Stroop procedure used^13,20^ comprised the sequential presentation of a fixation cross, a cue and a color-word stimuli which were displayed on a black screen background. The white fixation cross appeared at the center of the screen for 1000 ms, followed by a symbolized cue (continuous line for “read the written word” and dashed line for “name the ink color”) presented for 300 ms and indicating which stimulus feature (written word or ink color) represented the appropriate response for the color-word stimulus. Following the offset of the cue, the screen was blank for 1500 ms. Color-word stimuli were made up of the words RED, BLUE and GREEN and the ink colors were mapped to three keys on a computer keyboard. In the congruent condition, the written color names were printed in their respective ink colors (i.e., word RED printed in red ink, word BLUE printed in blue ink, and word GREEN printed in green ink). In the incongruent condition, the written color name was printed in one of the two remaining ink colors (e.g., word RED printed in green or blue ink color) providing six different combinations for incongruent trials. The color-word stimulus was presented on the screen until the individual made a response. Immediately following the response, an inter-trial blank screen was presented for 1000 ms, followed by the white fixation cross indicating the beginning of the next trial.

A total of 324 trials were presented, divided into 3 blocks of 108 trials each. Each block, comprising congruent and incongruent trials (half preceded by a cue indicating to identify the ink color and half by a cue indicating to identify the written word), was presented in a random order.

### Analysis of behavioral data

Response time and accuracy of correct responses were subjected to a repeated-measures analysis of variance (ANOVA) with task (word-reading, color-naming) and congruency (congruent, incongruent) as within-subject factors. A Greenhouse-Geisser correction was applied to avoid the violation of the sphericity assumption. Pairwise comparisons were used to interpret significant interactions.

### EEG recording and ERP analysis

Continuous EEG was recorded from 64 electrodes (Biosemi ActiveTwo system) placed at the international 10–20 system location and two pairs of bipolar electrodes recorded EOG in both vertical and horizontal directions. Two additional electrodes (active CMS: common mode sense, and passive DRL: driven right leg) were used as reference and ground to compose a feedback loop for amplifier reference. All data were digitized at 1024 Hz with 24-bit A/D conversion and combined to stimulus delivery and response recording monitored using E-Prime 2 (Psychology Software Tools, Pittsburgh, Pa., USA) and automatically synchronized with markers in the continuous EEG file. EEG signals were pre-processed with Brain Vision Analyzer (Version 2.0.1.391; Brain Products). Data were band-pass filtered between 0.3 Hz and 30 Hz using a zero-phase shift second-order Butterworth filter and vertical and horizontal eye movement artifacts were corrected by using an independent component analysis^57^ (ICA). ERPs were computed using a time window from the onset of the color-word stimulus to 1000 ms after. In addition to an automatic artifact rejection applying criteria of maximal voltage step of 50 μV, a maximal difference of values in intervals of 200 ms of 150 μV, and a maximal amplitude of ±100 μV, data were screened manually for residual artifacts. Channels containing excessive noise were replaced by using linear splines interpolation of adjacent electrodes^58^. Data were then re-calculated to an average reference^49^ and individual artifact-free ERPs were computed for each experimental condition by averaging corresponding trials with a correct response. After pre-processing, the mean percentage of remaining trials (mean ± standard deviation) was 80 ± 10 for the wC, 75 ± 9 for the wI, 81 ± 9 for the cC, and 73 ± 9 for the cI condition, respectively. No baseline correction was applied.

#### Consistency between subjects and microstate analysis

A topographic consistency test (TCT), based on non-parametric randomization techniques and implemented in the open-source software RAGU^52^ (Randomization Graphical User interface) based on Matlab (http://www.mathworks.com/), was applied to the ERPs of each experimental condition to identify the time points in which there is positive evidence for a consistent pattern of active sources across subjects^59^. This test allows selecting time periods where there is evidence for a consistent set of neuronal sources linked to the studied event that can then be used for further analysis. The TCT was computed with 10’000 randomization runs, and a *p* threshold of 0.01 (for a detailed description of this procedure see section SI.1 in the Supplementary Information File). To protect the TCT test from false positive caused by multiple testing, additional testing based on the duration of continuous periods of significance observed in our data was performed^52,59,60^ (for a detailed description of this procedure see section SI.3 in the Supplementary Information File). This was done to test if the duration of a significant time period exceeded chance.

Dynamic changes in ERP topographies were classified through microstate analysis. Microstate analysis decomposes the ERP signal into a set of stable topographical map configurations (microstates). This analysis assumes that stable brain functional states may vary between conditions in their duration and strength of activation^49,50,61^. In our study, microstate clustering was computed using the algorithm implemented in RAGU^53^ (for a detailed description of this procedure see section SI.4 in the Supplementary Information File). Essentially, the algorithm first identifies the optimal number of microstate prototype maps though a cross-validation procedure, an iterative technique requiring randomly splitting the available ERPs into learning and test sets, and then determines their topographical patterns. The cross-validation procedure was applied 250 times to our dataset, each time randomly dividing the 38 subjects into training and learning datasets of 19 subjects each, testing between 3 and 20 microstate classes. The k-means algorithm with 50 random initializations^62^ was used to identify each microstate map. Final microstate maps were computed using the grand average ERPs of all available subjects and conditions.

Values of the onset, offset, duration, and area under the curve (AUC) of each microstate map were extracted from the fitting of the microstate maps on the grand mean ERPs of each experimental condition. The onset and offset represent the beginning and the end time of a given microstate, while the duration represents the difference between offset and onset time. The AUC (ms x µV) was used to measure the amount of activation of a given microstate map and corresponds to the product of the duration and the global field power (GFP). The GFP is a global and reference independent measure of scalp field strength^49,51^ and is mathematically defined as the root mean square (RMS) across the average-references electrode values at a given instant of time.

Statistical analysis of microstate parameters was performed using randomization statistics^53^ (for a detailed description of this procedure see the section SI.5 in the Supplementary Information File). In our work, statistical analysis was performed with task (word-reading, color-naming) and congruency (congruent, incongruent) as within-subject factors, 10’000 randomization runs, and a *p* threshold of 0.01.

#### Analysis of topographical differences

Complementary to the microstate analysis described above, and to assess qualitative topographic differences between conditions in a constant time period^50,51,63,64^, we used a topographic analysis of variance (TANOVA); that is, a non-parametric randomization test based on global dissimilarities between electric fields. In contrast to channel-wise comparisons, the TANOVA computes global dissimilarity of the whole electrical field topographies between conditions and test for the significance of these topographic differences at each time point.

The TANOVA was implemented on the amplitude-normalized maps (GFP = 1), such that the results obtained are independent of the global field strength. The rationale behind this approach is that significant differences between conditions can be attributed to partially different sources of the evoked potential, and not to different strength of similar source distributions. The TANOVA was computed with the open-source software RAGU^52^ at each time point with task (word-reading, color-naming) and congruency (congruent, incongruent) as within-subject factors, with 10’000 randomization runs, and a *p* threshold of 0.01 (for a detailed description of this procedure see section SI.2 in the Supplementary Information File). To protect the TANOVA results from false positive caused by multiple testing, additional testing based on the duration of continuous periods of significance observed in our data was performed^52,59,60^ (for a detailed description of this procedure see section SI.3 in the Supplementary Information File). This was done to test whether the duration of a significant time period exceeded chance. After observing periods above duration threshold, post-hoc channel-wise *t*-test (*t*-maps) enabled further investigation of the topographic distribution of the observed differences.

Comparison between significant time periods revealed by the TANOVA analysis and the results obtained from the microstate analysis was used to clarify the nature of the topographic differences. In particular, whether the observed differences were due to latency shifts in the microstate maps occurrence caused by increased duration of certain microstate maps, or due to the presence of additional microstate maps in one of the experimental conditions. Moreover, given its sensitivity, TANOVA could also reveal time periods with relevant topographic differences between experimental conditions not detected by the microstate analysis.

#### Source localization

To determine differences in the pattern of neural generator activation responsible for the topographic differences observed between experimental conditions, a standardized low-resolution electromagnetic tomography method^54,65^ (sLORETA) was used. The sLORETA is a linear inverse imaging method that is obtained by standardizing a minimal norm inverse solution by source variance and measurement noise (see Pascual-Marqui^54^ for further details). The sLORETA solution space corresponds to 6239 voxels at 5 mm spatial resolution, restricted to grey matter of cortical and hippocampus regions according to the digitized atlas of the Montreal Neurological Institute. Voxel-wise *t* tests on the normalized current density data were performed for the timeframes and conditions displaying significant topographical differences, as highlighted by previous microstate and TANOVA analyses. Cortical voxels were identified as statistically different though a non-parametric randomization test^66^ with 5’000 permutations. This procedure determined the critical probability threshold (*p* < 0.05, one-tailed) for the observed *t*-values with correction for multiple testing.

## Results

### Behavioral data

The averages for response time and correct response (accuracy) were subjected to repeated measures ANOVAs. The average response time was significantly faster in the congruent (753.7, SE = 34.6) as compared to the incongruent (925.6, SE = 45.1) condition (F(1,37) = 84.49, p < 0.001, η^2^ = 0.69), and the average of accuracy was significantly higher in the congruent (mean = 0.969, SE = 0.42) than in the incongruent (mean = 0.905, SE = 0.69) condition (F(1,37) = 102.28, p < 0.001, η^2^ = 0.73). Both analyses revealed a significant interaction between task and condition. With respect to the average response time, the significant interaction observed between these two factors (F(1,37) = 12.40, p < 0.001, η^2^ = 0.25) was due to a faster average speed of response in the cC (mean = 725.5, SE = 33.5) as compared to wC (mean = 781.8, SE = 36.8) conditions (F(1,37) = 18.87, p < 0.001, η^2^ = 0.34). With respect to the average accuracy, the significant interaction observed between these two factors (F(1,37) = 6.91, p = 0.012, η^2^ = 0.16) was due to a higher accuracy in cC condition (mean = 97.46, SE = 0.51) as compared to wC (mean = 96.44, SE = 0.46) condition (F(1,37) = 4.4, p = 0.043, η^2^ = 0.1) and in wI condition (mean = 91.78, SE = 0.88) as compared to cI (mean = 89.32, SE = 0.91) condition (F(1,37) = 4.65, p = 0.037, η^2^ = 0.1). No main effect of task was observed in both the average response time (F(1,37) = 1.44, p = 0.242) and accuracy (F(1,37) = 1.56, p = 0.223).

### TCT and microstate analysis

**Figure 1:**
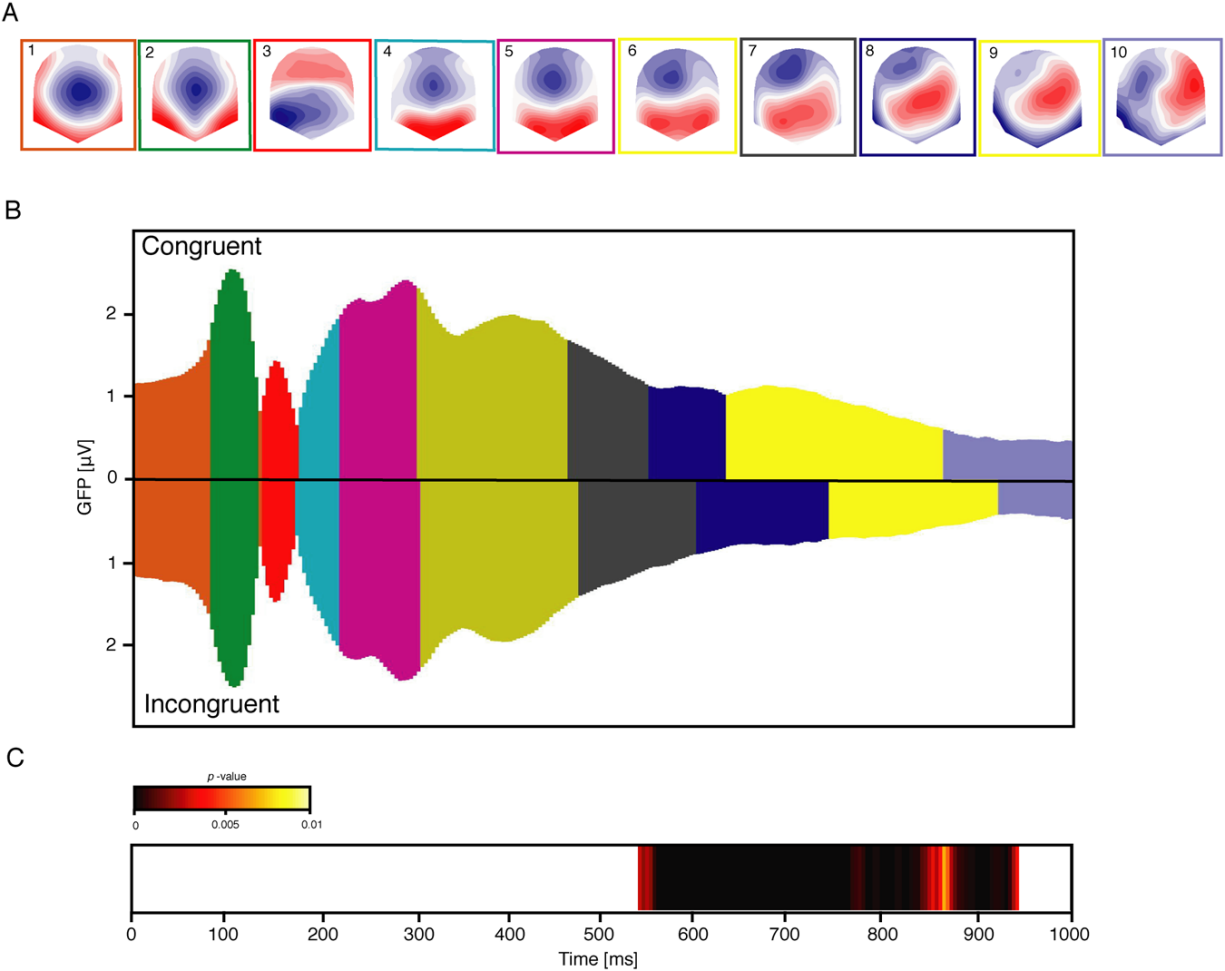
Microstate analysis and TANOVA results outlining periods of topographic differences of the grand mean ERPs of congruent and incongruent conditions. (**A**) Topographies of the ten microstate maps obtained from the cross-validation procedure. The microstate maps are labelled from 1 to 10 and displayed in sequence of occurrence from left to right. Red and blue indicate positive and negative potential values, respectively; all maps have been scaled to have GFP = 1. The colored frame surrounding each microstate map indicates the assignment shown in B. (**B**) The assignment of the ten microstate maps to the grand mean data of the congruent (upper) and incongruent (lower) condition as function of time (horizontal axis) and GFP (vertical axis). The colors of the areas refer to the colored frames surrounding each microstate maps shown in A. (**C**) The color-coded *p*-values of the TANOVA plotted for each time point. The white temporal windows reflect non-significant topographic differences, while the yellow-to-back window highlights significantly differing topographies (*p* < 0.01).

The TCT was applied on the pre-processed ERPs computed from the onset of color-word stimulus (0 ms) to 1000 ms after. The test showed significant consistent topographies over the entire period (from 0 to 1000 ms), corroborated by a significant global duration test performed on the duration of the significant time period. Microstate analysis was thus performed over the entire period. The cross-validation procedure identified an optimum of ten microstate maps (Fig. 1A) that were used for subsequent analyses.

The fitting of the microstate maps to the grand-mean ERP between 0 and 1000 ms resulted in the same ten microstate maps and the same temporal sequence in each experimental condition. Because none of the maps identified was exclusively related to any of the experimental conditions, possible differences in the duration of the microstates were further explored. Randomization statistics performed on the duration of each of the ten microstate maps neither revealed significant task effects nor significant interactions between task and congruency. This analysis revealed, however, a significant effect of congruency in the duration of microstate map 7 (*p* < 0.001) and microstate map 8 (*p* = 0.002) that was examined in more detail.

The chain of processes from stimulus perception to the manual response is illustrated in Fig. 1A,B. As the onset of microstate map 10 occurred approximately after the average response time in both the congruent and incongruent conditions, it was excluded from further analysis. Table 1 shows the details of the onset, duration and offset of each microstate map in both congruent and incongruent conditions as well as the significance of the differences obtained from the randomization statistics on microstate map parameters. These results indicated a temporal shift between congruent and incongruent conditions on-setting with the occurrence of microstate map 7. Specifically, an increased duration of microstate map 7 and microstate map 8 was observed in the incongruent condition. Microstate map 9 occurred later in the incongruent condition, as revealed by an increased onset and offset time in this condition. Finally, the analyses of the AUC revealed a significant main effect of congruency only for microstate map 9, with a higher AUC in the congruent condition (215 ms × μV) as compared to the incongruent (113 ms × μV) condition (*p* = 0.001). No other significant effects (task or task by congruency interaction) were observed in any of the AUC of the considered microstate maps.

**Table 1:** Descriptive onset, offset and duration (in milliseconds) of microstate maps in the congruent and incongruent condition.

Microstate	Parameter	Congruent	Incongruent	*p*-value
Map 1	Onset [ms]	0	0	ns
	Duration [ms]	86	86	ns
	Offset [ms]	86	86	ns
Map 2	Onset [ms]	86	86	ns
	Duration [ms]	55	55	ns
	Offset [ms]	141	141	ns
Map 3	Onset [ms]	141	141	ns
	Duration [ms]	39	35	ns
	Offset [ms]	180	176	ns
Map 4	Onset [ms]	180	176	ns
	Duration [ms]	43	47	ns
	Offset [ms]	223	223	ns
Map 5	Onset [ms]	223	223	ns
	Duration [ms]	82	86	ns
	Offset [ms]	305	309	ns
Map 6	Onset [ms]	305	309	ns
	Duration [ms]	160	167	ns
	Offset [ms]	465	476	ns
Map 7	Onset [ms]	465	476	ns
	Duration [ms]	86	126	*p* < 0.001
	Offset [ms]	551	602	*p* < 0.001
Map 8	Onset [ms]	551	602	*p* < 0.001
	Duration [ms]	82	140	*p* = 0.002
	Offset [ms]	633	742	*p* < 0.001
Map 9	Onset [ms]	633	742	*p* < 0.001
	Duration [ms]	230	180	ns
	Offset [ms]	863	922	*p* = 0.01

Also shown are the *p*-values for the congruency effect resulting from the microstate parameters statistical analysis. “ns” indicates a not significant effect.

### Topographical analysis and source localization

Differences in ERP topography between experimental conditions were examined using the TANOVA analysis. Within a time period ranging from 538 to 939 ms, the TANOVA revealed significant differences between congruent and incongruent topographies (Fig. 1C; for a time course representation of the ERPs see Supplementary Fig. S1), corroborated by a significant global duration test performed on the duration of the significant time period. This time period also corresponded to the differences observed between microstate map duration in the congruent and incongruent conditions occurring between 551 ms (offset of microstate map 7 in the congruent condition) and 922 ms (offset of microstate map 9 in the incongruent condition) (Fig. 1B and Table 1). This similarity indicated that the topographic differences revealed by the TANOVA were compatible with altered latencies of the underlying ERP components.

**Figure 2:**
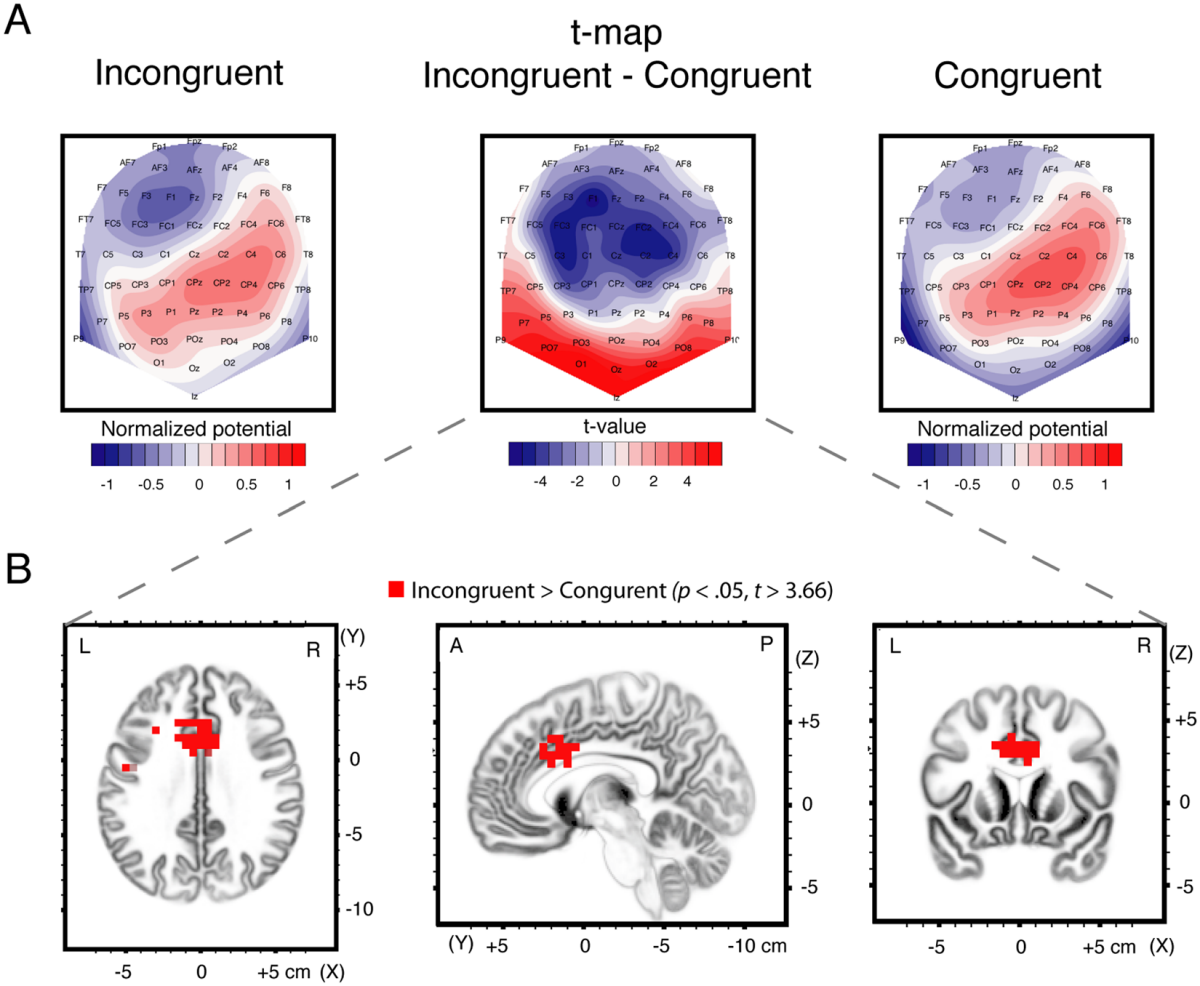
*t*-map and sLORETA contrasts between congruent and incongruent conditions computed in the time period between 538 and 742 ms. (**A**) Mean ERP mean topographies computed for the congruent (left) and incongruent (right) conditions. Topographies were normalized (GFP = 1). Red and blue indicate positive and negative potential values, respectively. Also shown in the middle is a standard *t*-map contrasting incongruent and congruent topographies in the same time period. Positive (red) and negative (blue) *t*-values indicate more positive and more negative potential in the incongruent condition compared to the congruent condition, respectively. (**B**) Voxel-wise *t* values comparing the sLORETA source density between the incongruent and congruent condition. Cluster of voxels located in the anterior cingulate cortex (BA 24 and 33) and cingulate gyrus (BA 24 and 32) showed increased activation in the incongruent compared to the congruent condition. All voxels reaching the threshold for *p* < 0.05 (corrected for multiple comparison, *t* > 3.66) are color-coded in red.

To quantify these latency effects, topographical differences between the congruent and incongruent conditions were further examined between 538 and 742 ms. The onset of this selected time window corresponded to the initial time of observed topographic differences, as outlined by the TANOVA (Fig. 1C). The offset was chosen to coincide with the offset time of microstate map 8 in the incongruent condition. Post-hoc channel-wise *t*-test (*t*-maps) within this time period contributed to a better understanding of the topographic distribution of these differences, and thus put into perspective our findings with the existing EEG studies. The average topographies obtained in the selected time window for the congruent and incongruent conditions together with the *t*-map contrast between these conditions are shown in Fig. 2A. The *t*-map contrasts revealed that the incongruent condition was characterized by a more negative potential over frontal-central electrodes and a more diffused positive potential localized over posterior and occipital regions (t_max_ = 6.58 at electrode F1; t_min_ = −5.1 at electrode O1).

sLORETA was applied in the same temporal window (538 ms to 742 ms) to approximate brain regions responsible for the observed topographic difference between congruent and incongruent ERPs. The statistical analysis revealed a cluster of 41 adjacent voxels (*t* > 3.66 for a *p* < 0.05) that differed in current density estimates between congruent and incongruent conditions (Fig. 2B). This cluster, localized bilaterally in the ACC (BA 24 and 33) and in adjacent regions of the cingulate gyrus (BA 24 and 32), showed significantly higher activity in the incongruent condition as compared to the congruent condition. The maximal *t*-value of the cluster (*t* = 4.18, *p* = 0.012) was located in the BA 24 (Talairach coordinates x = −10, y = 16, z = 27).

**Figure 3:**
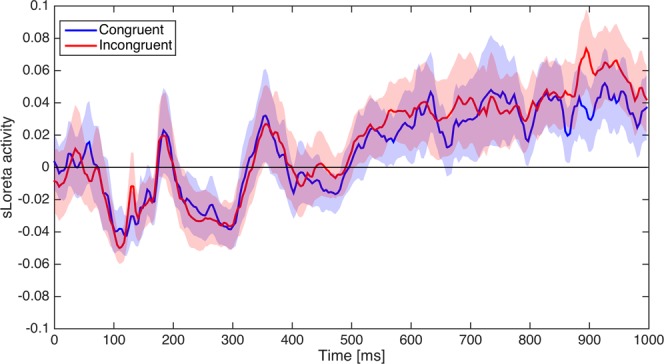
Dynamics of the global ACC activity obtained from the average of the sLoreta activity of 144 voxels located in the ACC (BA 10, 24, 25, 32 and 33) for the congruent (blue line) and incongruent (red line) condition, respectively. The blue and red shadowed areas represent the surface that encloses the Mean ± SE of the congruent and incongruent conditions, respectively.

Finally, an additional analysis was run in the source space with the overall objective of comparing the global strength of activity of the ACC between corresponding microstate maps in the congruent and incongruent condition. To this end, the time course of ACC activity from 0 ms to 1000 ms (Fig. 3) was first obtained in both experimental conditions by averaging current density data extracted from the corresponding voxels in the ACC region (144 voxels in total, covering BA 10, 24, 25, 32, 33). This configuration can be considered as the equivalent of the activity of a generator situated in the ACC. The time course of ACC activity displayed in Fig. 3 suggests an increase of activity on-setting approximately around 500 ms in both conditions.

The resulting activity was then compared between congruent and incongruent conditions across nine different time periods defined by the onset and offset time of the respective microstate map in each experimental condition. More specifically, for each subject the activity was averaged according to each condition and each time period (e.g., for the 8^th^ time period, the ACC activity was averaged according to the onset and offset time of microstate map 8, that is between 551 and 633 ms in the congruent condition and between 602 and 742 ms in the incongruent condition). To evaluate the null-hypothesis of no difference in microstate maps strength of activity between experimental conditions with the alternative hypothesis of a difference in strength of activity between experimental conditions, a Bayesian paired samples *t-*test^67,68^ was run separately for each time period. The estimated Bayes factors BF_01_ (Table 2), comparing the fit of the data under the null hypothesis and the alternative hypothesis, suggested that our data displayed a moderate evidence for an absence of difference (null hypothesis) in microstate maps strength of activity between congruent and incongruent conditions. In particular, the null-hypothesis model was always more than three times more likely than the alternative model.

**Table 2:** Values of the Bayes factor BF_01_ obtained from the Bayesian paired samples *t*-test comparing the average ACC source activity across the respective microstate map in each condition.

Map 1	Map 2	Map 3	Map 4	Map 5	Map 6	Map 7	Map 8	Map 9
**Bayes Factor BF**_**01**_								
5.12	5.68	5.71	5.63	5.21	5.25	3.36	3.71	3.74

## Discussion

The main objective of this work was to help disentangle the debate regarding activity of the ACC during conflict. The debate attempts to determine whether higher ACC activation specifically reflects a conflict between brain pathways processing distinct aspects of information^8^, or whether a longer duration of ACC activation reflects more generally the brain operations needed to select the correct response^43,45–47^. To this end, we have combined microstate, topographic, and source localization analyses to fully exploit the spatiotemporal information from EEG recordings of participants performing a Stroop paradigm.

The behavioral results observed in the Stoop task reflected those already observed by MacDonald and colleagues^13^ and West and colleagues^20^ in a similar procedure. The interference effects induced by the competition between responses (word reading and color naming) resulted in slower RTs and increased errors in the incongruent condition as compared to the congruent condition.

The microstate analyses regard the time period between the stimulus onset and the motor response: the microstate analysis identified 10 maps occurring in the same temporal sequence and did not differ between congruent and incongruent conditions. Components underlying processes ranging from early visual perceptual (P1) to information processing (P3) were associated with microstate maps 1 to 6 in both the congruent and incongruent conditions. These results were consistent with those shown by previous Stroop EEG studies^20,33,35,36,38,55,69^. Fluctuations in the duration of ERP components were observed from microstate map 7 to microstate map 9. Statistical evaluation of microstate map parameters (onset, offset and duration) revealed a faster succession of states in the congruent condition than in the incongruent one. Microstate maps 7 and 8 lasted longer in the incongruent condition and both microstate maps 8 and 9 appeared later in time in the incongruent condition.

The topographical characteristics observed during the time period covered by microstate map 7 and microstate map 8 were a frontal-central negativity with a predominant posterior positivity moving towards the anterior right direction from microstate map 7 to microstate map 8 (and continuing in microstate map 9) comparable to previous findings^56,70^. The TANOVA yielded compatible and converging results with those of the microstate analysis, with topographic differences between conditions occurring concomitantly with time periods of observed microstate maps latency shifts (from 538 ms to 922 ms). Quantification of these differences (by contrasting incongruent and congruent conditions within a time window from 538 to 742 ms including microstate map 7 and microstate map 8) revealed a diffused frontal-central negativity, caused by a more negative electrical potential in the incongruent condition as compared to the congruent one. Finally, source estimation indicated that the brain region responsible for the topographic differences between congruent and incongruent ERPs was an area in the ACC (BA 24 and 33), and adjacent regions of the cingulate gyrus (BA 24 and 32), that was more active in the incongruent condition than in the congruent condition. These findings are compatible with previous EEG studies using a Stroop paradigm^20,33,35,36,38,55,69,71^. These studies consistently reported a more intense and diffused negative electrical potential over frontal-central regions in the incongruent as compared to the congruent conditions (N_inc_ component), and related these differences to neural generators localized in the ACC regions^20,35,72^. It should be noted that in our study the latency of the observed differences seemed to occur slightly later in time compared to previous works. This could be related to a higher average reaction time observed in our data in both the congruent and incongruent conditions.

Taken as a whole, our results contrast with the possibility of an additional brain process that would explain the longer RTs observed in the incongruent condition. Instead, our findings support the possibility that the same large neuronal networks are involved in information processing ranging from perception to response irrespective of the nature (congruent or incongruent) of the stimulus^56,63^.

The hypothesis of a functional specialization of the ACC in the detection of interference between alternative responses is based on a series of functional brain imaging findings^10,13^ indicating increased activation of the ACC in incongruent trials. This data provided the basis for the conflict-monitoring model^8^. This model was however questioned by a relevant argument pointing out the limited temporal resolution of fMRI techniques and the slow response of BOLD signal^47^: On the basis of fMRI alone, it is difficult to determine whether the increased activation observed in the literature that underlies the incongruent condition (compared to the congruent condition) is a result of higher neuronal activity per time unit, or a result of the same degree of processing but for a longer period of time^47^. In both cases (higher activity vs. longer activity), the recorded BOLD signal would show higher activation in the incongruent condition.

The results of the present study regard brain dynamics from the onset of the color-word stimuli until the release of the motor response. At the scalp level, the microstate and TANOVA analysis contradicted the presence of a unique additional microstate map in the incongruent condition as hypothesized by the conflict-monitoring model. Indeed, these analyses demonstrated that the electrical dynamics differed in duration toward the end of information processing, as showed by the increased duration of microstate map 7 and 8 in the incongruent condition. At the source level, the higher activation of an area in the ACC and adjacent regions of the cingulate gyrus in the incongruent condition could receive an interesting explanation based on the findings of the microstate and TANOVA analysis. More precisely, one plausible interpretation of the observed effects was that the activity of this portion of the ACC and the cingulate gyrus decreased earlier in the congruent condition. This suggests that, at this stage of processing, these regions are less necessary for the selection and release of the motor response in the congruent condition while they are still needed and active in the incongruent condition.

In accordance with the results obtained from our ERP data, it seems reasonable to think that the increase in activity of the ACC regions in the incongruent condition -commonly reported in various EEG and imaging studies- was not related to an increase of the amplitude of the ERPs, but to an increase of its duration. This interpretation is supported by an independent analysis based on Bayes factors and performed on the overall activity of the ACC region across microstate maps, which provided moderate statistical evidence in support to an equal strength of activity of the ACC between congruent and incongruent conditions (considered as a null hypothesis), within a time period ranging from the onset of the color-word stimuli to the release of the motor response.

From a general point of view, our findings are more in agreement with the hypothesis of a longer neuronal activity of the ACC in the incongruent condition as theorized by Grinband and colleagues^47^. By contrast, they give less support to the possibility of a conflict monitoring specificity of the ACC predicting a higher neuronal activity in the incongruent condition^8^. At this stage of knowledge, however, clarifying the change in the duration of activation remains an open issue. For the time being, we can only rely on the general assumption that it reflects all the mechanisms (sensory, memory, attention and motor processes) involved in releasing a response to competing stimuli^47^.

Overall, this work emphasizes the caution that must be used when interpreting fMRI results. Particularly, temporal brain dynamics can dramatically change through periods of interest, and fMRI is limited in its ability to access and assess these temporal changes. The current work also highlights that using measures sensitive to the temporality of events to explore the brain mechanisms can contribute to a better understanding of the mechanisms underlying information processing, and thus offers new venues to explore conflict monitoring.

Finally, our findings, issued from the use of complementary methods including microstate and TANOVA analyses, are linked to the specificity of our data set. Thus, we cannot exclude the possibility that, under different conditions (e.g., with another independent data set, with more subjects added to the analysis, with a higher ERPs’ signal-to-noise ratio or with a different design of the Stroop task), the explanation based on a higher ACC activation would be the most appropriate.

## Supplementary information

Supplementary information

## References

[1] Norman DA (1968). Toward a theory of memory and attention. Psychol. Rev..

[2] Gratton G, Cooper P, Fabiani M, Carter CS, Karayanidis F (2018). Dynamics of cognitive control: Theoretical bases, paradigms, and a view for the future. Psychophysiology.

[3] Miyake A, Friedman NP (2012). The Nature and Organisation of Individual Differences in Executive Functions: Four General Conclusions. Curr. Dir. Psychol. Sci..

[4] Miyake A (2000). The Unity and Diversity of Executive Functions and Their Contributions to Complex ‘Frontal Lobe’ Tasks: A Latent Variable Analysis. Cogn. Psychol..

[5] MI Posner SP (1990). -1989. The attention system of the human brain. Annu. Rev. Neuroscince.

[6] Curtis CE, D’Esposito M (2003). Persistent activity in the prefrontal cortex during working memory. Trends Cogn. Sci..

[7] Botvinick M, Nystrom LE, Fissell K, Carter CS, Cohen JD (1999). Conflict monitoring versus selection-for-action in anterior cingulate cortex. Nature.

[8] Botvinick MM, Braver TS, Barch DM, Carter CS, Cohen JD (2001). Conflict Monitoring and Cognitive Control Despite the importance of these efforts to characterize the func- tion of cognitive control, most of them share an important limita- tion in scope. Most current theories focus nearly exclusively on the. Psychol. Rev..

[9] Botvinick MM, Cohen JD, Carter CS (2004). Conflict monitoring and anterior cingulate cortex: An update. Trends Cogn. Sci..

[10] Carter CS (1998). Anterior cingulate cortex, error detection, and the online monitoring of performance. Science (80-.)..

[11] Carter CS (2000). Parsing executive processes: Strategic vs. evaluative functions of the anterior cingulate cortex. Proc. Natl. Acad. Sci..

[12] Kerns JG (2004). Anterior cingulate conflict monitoring and adjustments in ntrol.co. Science.

[13] Macdonald AW (2000). And Anterior Cingulate Cortex in Cognitive Control Dissociating the Role of the Dorsolateral Prefrontal and Anterior Cingulate Cortex in Cognitive Control. Science (80-.)..

[14] Van Veen V, Cohen JD, Botvinick MM, Stenger VA, Carter CS (2001). Anterior cingulate cortex, conflict monitoring, and levels of processing. Neuroimage.

[15] Casey BJ (2000). Dissociation of response conflict, attentional selection, and expectancy with functional magnetic resonance imaging. Proc. Natl. Acad. Sci..

[16] Stroop, J. R. Studies of interference in serial verbal reactions. *J Exp Psychol*. **18**(6), 643–62 (1935).

[17] MacLeod CM (1991). Half a century of research on the Stroop effect: an integrative review. Psychol. Bull..

[18] Eriksen BA, Eriksen CW (1974). Effects of noise letterls upon the identification of a target letter in a non-search task. Percept. Psychophys..

[19] Scerrati E, Lugli L, Nicoletti R, Umiltà C (2017). Comparing Stroop-like and Simon Effects on Perceptual Features. Sci. Rep..

[20] West R (2003). Neural correlates of cognitive control and conflict detection in the Stroop and digit-localisation tasks. Neuropsychologia.

[21] Pardo JV, Pardo PJ, Janer KW, Raichle ME (1990). The anterior cingulate cortex mediates processing selection in the Stroop attentional conflict paradigm. Proc. Natl. Acad. Sci..

[22] Carter. Interference and Facilitation Effects during Selective Attention.pdf (1995). 10.1006/nimg.1995.1034 9343611

[23] Bench CJ (1993). Investigations of the funcitonal anatomy of attention using the Stoop test. Neuropsychologia.

[24] George MS (1994). Regional Brain Activity When Selecting a Response green blue black griet misery sad bleak. Hum. Brain Mapp..

[25] Banich M (2000). fMri studies of Stroop tasks reveal unique roles of anterior and posterior brain systems in attentional selection. J. Cogn. Neurosci..

[26] Milham MP, Banich MT (2005). Anterior Cingulate Cortex: An fMRI Analysis of Conflict Specificity and Functional Differentiation. Hum. Brain Mapp..

[27] Van Veen V, Carter CS (2002). The anterior cingulate as a conflict monitor: FMRI and ERP studies. Physiol. Behav..

[28] Van Veen V, Carter CS (2002). The timing of action-monitoring processes in the anterior cingulate cortex. J. Cogn. Neurosci..

[29] Milham MP (2001). The relative involvement of anterior cingulate and prefrontal cortex in attentional control depends on nature of conflict. Cogn. Brain Res..

[30] Milham MP, Banich MT, Claus ED, Cohen NJ (2003). Practice-related effects demonstrate complementary roles of anterior cingulate and prefrontal cortices in attentional control. Neuroimage.

[31] Bush G (1998). The counting Stroop: an interference task specialized for functional neuroimaging–validation study with functional MRI. Hum. Brain Mapp..

[32] Durston S (2003). Parametric manipulation of conflict and response competition using rapid mixed-trial event-related fMRI. Neuroimage.

[33] West R, Alain C (1999). Event-related neural activity associated with the Stroop task. Brain Res. Cogn. Brain Res..

[34] West R, Alain C (2000). Effects of task context and fluctuations of attention on neural activity supporting performance of the Stroop task. Brain Res..

[35] Liotti M, Woldorff MG, Perez R, Mayberg HS (2000). An ERP study of the temporal course of the Stroop color-word interference effect. Neuropsychologia.

[36] Larson MJ, Kaufman DAS, Perlstein WM (2009). Neural time course of conflict adaptation effects on the Stroop task. Neuropsychologia.

[37] Markela-Lerenc J (2004). Prefrontal-cingulate activation during executive control: Which comes first?. Cogn. Brain Res..

[38] Badzakova-Trajkov G, Barnett KJ, Waldie KE, Kirk IJ (2009). An ERP investigation of the Stroop task: The role of the cingulate in attentional allocation and conflict resolution. Brain Res..

[39] Coderre E, Conklin K, Van Heuven WJB (2011). Electrophysiological measures of conflict detection and resolution in the Stroop task. Brain Res..

[40] Macdonald JSP, Mathan S, Yeung N (2011). Trial-by-trial variations in subjective attentional state are reflected in ongoing prestimulus EEG alpha oscillations. Front. Psychol..

[41] Rebai M, Bernard C, Lannou J (1997). The Stroop’s Test Evokes A Negative Brain Potential, the N400. Int. J. Neurosci..

[42] Appelbaum LG, Boehler CN, Davis L, Won JR, Woldorff MG (2014). The Dynamics of Proactive and Reactive Cognitive Control Processes in the Human Brain. J. Cogn. Neurosci..

[43] Carp J, Fitzgerald KD, Taylor SF, Weissman DH (2012). Removing the effect of response time on brain activity reveals developmental differences in conflict processing in the posterior medial prefrontal cortex. Neuroimage.

[44] Yeung N, Cohen JD, Botvinick MM (2011). Errors of interpretation and modeling: A reply to Grinband *et al*. Neuroimage.

[45] Grinband JC (2011). error likelihood, and RT: Response to Brown & Yeung *et al*. Neuroimage.

[46] Grinband J, Wager TD, Lindquist M, Ferrera VP, Hirsch J (2008). Detection of time-varying signals in event-related fMRI designs. Neuroimage.

[47] Grinband J (2011). The dorsal medial frontal cortex is sensitive to time on task, not response conflict or error likelihood. Neuroimage.

[48] McKay CC, van den Berg B, Woldorff MG (2017). Neural cascade of conflict processing: Not just time-on-task. Neuropsychologia.

[49] Lehmann D, Skrandies W (1980). Reference-free identification of components of checkerboard-evoked multichannel potential fields. Electroencephalogr. Clin. Neurophysiol..

[50] Murray MM, Brunet D, Michel CM (2008). Topographic ERP analyses: A step-by-step tutorial review. Brain Topogr..

[51] Lehmann. Principles of spatial analysis. *Methods Anal. brain Electr. Magn. signals. EEG Handb*. 309–354 at, https://ci.nii.ac.jp/naid/10008474181/ (1987).

[52] Koenig T, Kottlow M, Stein M, Melie-García L (2011). Ragu: A free tool for the analysis of EEG and MEG event-related scalp field data using global randomization statistics. Comput. Intell. Neurosci..

[53] Koenig T, Stein M, Grieder M, Kottlow M (2014). A tutorial on data-driven methods for statistically assessing ERP topographies. Brain Topogr..

[54] Pascual-Marqui RD (2002). Standardized low-resolution brain electromagnetic tomography (sLORETA): technical details. Methods Find. Exp. Clin. Pharmacol..

[55] Khateb A, Michel CM, Pegna AJ, Landis T, Annoni J-M (2000). New insights into the Stroop effect: a spatio-temporal analysis of electric brain activity. Neuroreport.

[56] Schiller B (2016). Clocking the social mind by identifying mental processes in the IAT with electrical neuroimaging. Proc. Natl. Acad. Sci..

[57] Cardoso J-F (1998). Blind signal separation: statistical principles. Proc. IEEE.

[58] Perrin F, Pernier J, Bertrand O, Giard MH, Echallier JF (1987). Mapping of scalp potentials by surface spline interpolation. Electroencephalogr. Clin. Neurophysiol..

[59] Koenig T, Melie-García L (2010). A method to determine the presence of averaged event-related fields using randomization tests. Brain Topogr..

[60] Habermann M, Weusmann D, Stein M, Koenig T (2018). A student’s guide to randomization statistics for multichannel event-related potentials using Ragu. Front. Neurosci..

[61] Lehmann, D. In *Machinery of the Mind* 209–224 Birkhäuser Boston, 10.1007/978-1-4757-1083-0_10 (1990)

[62] Pascual-Marqui RD, Michel CM, Lehmann D (1995). Segmentation of brain electrical activity into microstates: model estimation and validation. IEEE Trans. Biomed. Eng..

[63] Michel, C. M. *Electrical neuroimaging*. Cambridge University Press, at, http://www.cambridge.org/ch/academic/subjects/medicine/medical-imaging/electrical-neuroimaging?format=HB&isbn=9780521879798#qYAOTwefzkKQP9ZW.97 (2009).

[64] Strik WK, Fallgatter AJ, Brandeis D, Pascual-Marqui RD (1998). Three-dimensional tomography of event-related potentials during response inhibition: evidence for phasic frontal lobe activation. Electroencephalogr. Clin. Neurophysiol..

[65] Pascual-Marqui RD (1999). Review of Methods for Solving the EEG Inverse Problem. Int. J. Bioelectromagn..

[66] Nichols TE, Holmes AP (2002). Nonparametric permutation tests for functional neuroimaging: a primer with examples. Hum. Brain Mapp..

[67] Wagenmakers E (2007). Wagenmakers, 2007 (PBR). Psychon. Bull. Rev..

[68] Harms, C. & Lakens, D. Making ‘Null Effects’ Informative: Statistical Techniques and Inferential Frameworks, 10.17605/OSF.IO/WPTJU (2018). PMC6412612 30873486

[69] West R, Jakubek K, Wymbs N, Perry M, Moore K (2005). Neural correlates of conflict processing. Exp. Brain Res..

[70] Egenolf Y (2013). Tracking the implicit self using event-related potentials. Cogn. Affect. Behav. Neurosci..

[71] Jiang J, Zhang Q, Van Gaal S (2015). EEG neural oscillatory dynamics reveal semantic and response conflict at difference levels of conflict awareness. Sci. Rep..

[72] Hanslmayr S (2008). The Electrophysiological Dynamics of Interference during the Stroop Task. J. Cogn. Neurosci..

