# Supplementary material for: A fine-grained time course investigation of brain dynamics during conflict monitoring
Source: Sci Rep. 2019 Mar 6;9:3667. doi: 10.1038/s41598-019-40277-3 (PMC6403345; doi:10.1038/s41598-019-40277-3)
Supplement: Supplementary file 1 — Supplementary information [file 41598_2019_40277_MOESM1_ESM.pdf]

Supplementary Information

A fine-grained time course investigation of brain dynamics during conflict monitoring

Paolo Ruggeri*[1], Hadj Boumediene Meziane[1], Thomas Koenig[2] and Catherine Brandner[1]

[1] Brain Electrophysiology Attention Movement Laboratory, Institute of Psychology, University of Lausanne, Switzerland.

[2] Translational Research Center, University Hospital of Psychiatry, University of Bern, Switzerland

SI METHODS

SI.1 Topographic consistency test

The TCT is based on the argument that the GFP of the grand mean ERP across subjects at one moment in time depends on both the GFP of the individual ERPs and on the consistency of the topography over subjects. Indeed, a low consistency of the topography across subjects will result in a GFP of the grand mean that is consistently lower than the mean of the individual GFP values. On the contrary, a high consistency of the topography across subjects will result in a GFP of the grand mean that is slightly lower than the mean of the individual GFP values. Thus, the TCT uses the GFP

of the grand mean as a measure of effect size (i.e., a measure of consistency of the topography across subjects).

For a given dataset, to test whether a given effect size may also have been observed by chance, the assumed structure of the data is destroyed by shuffling the measured potentials across electrodes in each individual ERP map. This procedure destroys the consistency of topography across subjects but preserves the GFP of the individual map. The null hypothesis is thus that the GFP of the grand mean before shuffling is about equally large as after shuffling. The null hypothesis can be rejected if the GFP of the grand mean is consistently larger when the channels are in correct order as opposed to when they are in randomized order. In other words, the probability of the null hypothesis is defined as the number of randomization runs yielding a GFP larger than or equal to the GFP obtained with the correct channel order. This procedure can be repeated for each ERP time point and separately for each experimental condition, resulting in a moment by moment test of map consistency.

SI.2 Topographic analysis of variance

The TANOVA tests for significant differences in topography between conditions. As a measure of effect size, the TANOVA uses the strength of the difference maps between conditions (i.e., the GFP of the difference maps). For simplicity, we describe in the following the randomization tests for topographical differences for the case of comparing two conditions (e.g., the congruent and incongruent conditions in our study): first, the scalp field maps are averaged separately for both conditions yielding two condition-wise grand mean maps. The GFP of the difference between the condition-wise maps is used as indicator of the strength of the difference and serves as a measure of effect size. For the creation of instances of effect size under the null hypothesis, the underlying structure of the data is eliminated by randomly permuting the ERP data of each subject between conditions. Once this randomization has been done, the (random-)condition specific grand means ERPs can be re-computed, and the GFP of the difference map between the two grand means can be

computed again. This represents one observation of the effect size under the null hypothesis. In order to estimate how compatible the observed GFP of the difference map is with the null hypothesis, the computation of the GFP values under the null hypothesis is repeated many times. Finally, the GFP of the difference map obtained in the measured data is compared to the distribution of the GFP of the difference map under the null hypothesis. The probability that the observed GFP difference was obtained by chance is then defined as the percentage of observations where this GFP was smaller than or equal to the GFP of the randomly obtained difference maps. Such a test can be applied to each time point of the ERP trace.

SI.3 Global duration statistics

Time-point by time-point tests, such as the TCT and TANOVA, require methods of accounting for multiple testing over time. As described in the previous paragraphs, a test for significance can be computed for every time point for both the TCT and TANOVA, yielding a distribution of *p*-values: a subset of these will be below a chosen threshold by chance, and thus constitute false positive if this is the only criterion for significance. One approach to minimize this problem is to perform additional testing by quantifying the duration of contiguous periods with sub-threshold *p*-values. To estimate how likely it is that a given duration of sub-threshold *p*-values are present under the null-hypothesis, the previously obtained results from the randomization procedure are re-used. For every randomization run computed during the TCT (or the TANOVA), *p*-values can be computed by comparing the obtained random differences with those obtained in all other randomization runs. This enables extraction of the duration of periods of sub-threshold *p*-values that are expected under the null-hypothesis, yielding a distribution of the duration of false-positives under the null-hypothesis. Finally, the duration of contiguous sub-threshold *p*-values in the measured data can be compared to this distribution of duration of contiguous false positives in the randomized data. If, for example, a 1% *p*-threshold for the overall significance is chosen, then the output of the global duration statistics indicates the duration of periods of contiguous *p*-values that is larger than 99% of

the false positive duration of contiguous p-values obtained in the random data, and thus produces an overall 1% false positive rate.

SI.4 Selection of microstate maps number

The microstate clustering implemented in RAGU aims at identifying a microstate model that is sufficiently complex to accommodate the part of the data variance that is common across subjects, but not overly complex such that particularities of subsets of the sample are accounted for. This problem is addressed by the implementation of a microstate model selection through the following cross-validation procedure:

1. Subjects are divided into a training and test dataset.
2. Grand mean ERPs are computed in each dataset as a function of condition.
3. Microstate models with a different number of microstate maps are computed from the grand means of the training dataset. Each model contains both the topographies of the microstate maps and an index that assigns each time frame of the data to one of these microstate maps.
4. The mean spatial correlation of the test dataset with each microstate model is computed for each condition and time-point and averaged. The mean correlation corresponds to the explained variance of each microstate model with the test dataset.
5. Since the mean correlation of the test dataset with each microstate model depends on the division of the data into training and test datasets, steps 1-4 are repeated several times and the mean correlations from each run are retained.
6. The obtained mean correlations for each microstate model are averaged across repetitions. The mean correlation increases with increasing number of microstate maps and reaches a plateau after a certain number of microstate maps are considered in the microstate model. The chosen fitting number of microstate maps for the data is marked by the beginning of the plateau, because adding more microstate maps beyond this point does not add generalizable features to the microstate model and would results in an over-fitting of the data.

7. The microstate templates with the selected number of microstate maps are computed using the grand mean ERPs of all available subjects and conditions and used for the remainder of the analyses.

The microstate templates with the selected number of microstate maps can be therefore considered as a maximally complex representation of the generalizable features of the given dataset.

SI.5 Statistical analysis of microstate parameters

The goal of the microstate statistics is to evaluate ERP microstate features (e.g., onset, offset, duration, AUC) by comparing an effect (e.g., a difference in the onset of a given microstate map in the ERPs of two conditions) against the distribution of this effect under the null hypothesis. To this purpose, randomization statistics is used to determine this distribution based on simulations of the effect under the null hypothesis. To quantify an effect of interest in the measured data, microstate features are first obtained after the microstate maps have been assigned to the condition specific grand mean data. The quantifier of the effect of interest is then defined by the variance of the feature extracted from the different conditions. In the case of our dataset, for example, the quantifier of the effect of interest for the duration of a microstate map in the congruent and incongruent condition, could be defined as the difference in duration of the microstate map between the two conditions (the same logic can be used for the onset, offset and the AUC). In this case, for the creation of instances of the chosen quantifier under the null hypothesis, the underlying structure of the data is eliminated by randomly permuting the ERP data of each subject between congruent and incongruent conditions. Once this randomization has been done, the (random-) condition specific grand means ERPs can be computed, and the quantifier of interest (i.e. the variance of an ERP microstate feature across conditions) can again be computed as above. This represents one observation of the quantifier of interest under the null hypothesis. By performing multiple runs of the randomization procedure, a distribution of the quantifier of interest under the null hypothesis can be obtained. Finally, the quantifier obtained in the measured data is compared to the distribution

of the quantifier under the null hypothesis. This is done by rank statistics, where the probability of the data being compatible with the null hypothesis is defined by the proportion of quantifiers obtained under the null hypothesis that were larger or equal to the quantifier obtained from the measured data. Importantly, the distribution of the quantifier under the null hypothesis depends on the number of number of randomization runs. For a reliable rejection of the null-hypothesis on a 1% level, more than 5’000 randomization runs are recommended.

SI FIGURES

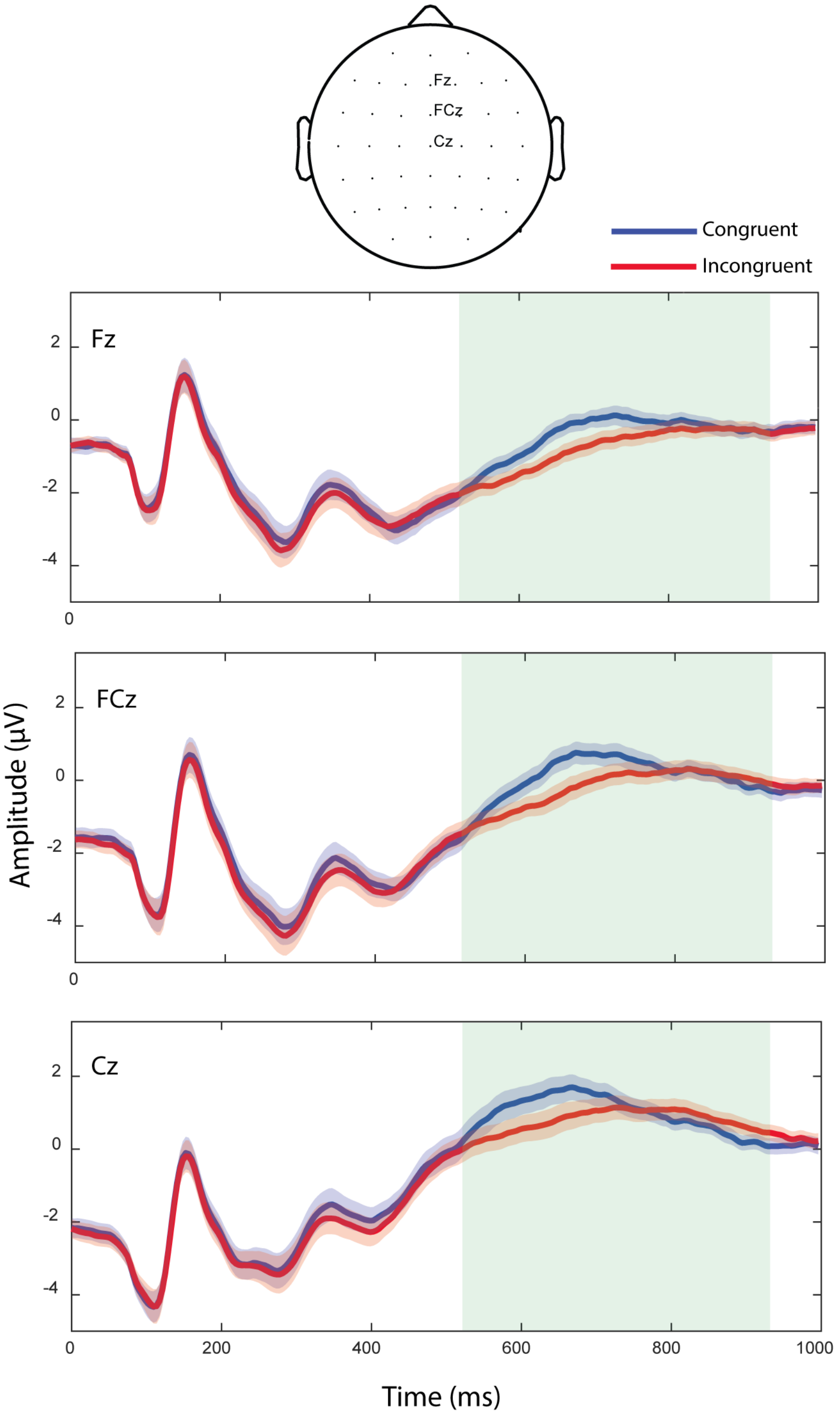

Fig. S1: ERP dynamics of frontal-central electrodes Fz, FCz and Cz for the congruent (blue line) and incongruent (red line) condition, respectively. The blue and red shadowed areas represent the surface that encloses the Mean ± SE of the congruent and incongruent conditions, respectively. The time period of significant differences between the congruent and incongruent conditions revealed by the TANOVA analysis (from 538 to 939 ms) is highlighted in green.
